# Effects of Embryonic Inflammation and Adolescent Psychosocial Environment on Cognition and Hippocampal Staufen in Middle-Aged Mice

**DOI:** 10.3389/fnagi.2020.578719

**Published:** 2020-09-11

**Authors:** Yong-Fang Wu, Yue-Ming Zhang, He-Hua Ge, Chong-Yang Ren, Zhe-Zhe Zhang, Lei Cao, Fang Wang, Gui-Hai Chen

**Affiliations:** ^1^Department of Neurology (Sleep Disorders), The Affiliated Chaohu Hospital of Anhui Medical University, Hefei, China; ^2^Department of Neurology and Critical Care, The First Affiliated Hospital of Anhui Medical University, Hefei, China; ^3^Department of Neurology, The Second Affiliated Hospital of Anhui Medical University, Hefei, China

**Keywords:** aging, inflammation, learning and memory, offspring, stress, Staufen

## Abstract

Accumulating evidence has indicated that embryonic inflammation could accelerate age-associated cognitive impairment, which can be attributed to dysregulation of synaptic plasticity-associated proteins, such as RNA-binding proteins (RBPs). Staufen is a double-stranded RBP that plays a critical role in the modulation of synaptic plasticity and memory. However, relatively few studies have investigated how embryonic inflammation affects cognition and neurobiology during aging, or how the adolescent psychosocial environment affects inflammation-induced remote cognitive impairment. Consequently, the aim of this study was to investigate whether these adverse factors can induce changes in Staufen expression, and whether these changes are correlated with cognitive impairment. In our study, CD-1 mice were administered lipopolysaccharides (LPS, 50 μg/kg) or an equal amount of saline (control) intraperitoneally during days 15–17 of gestation. At 2 months of age, male offspring were randomly exposed to stress (S), an enriched environment (E), or not treated (CON) and then assigned to five groups: LPS, LPS+S, LPS+E, CON, and CON+S. Mice were evaluated at 3-month-old (young) and 15-month-old (middle-aged). Cognitive function was assessed using the Morris water maze test, while Staufen expression was examined at both the protein and mRNA level using immunohistochemistry/western blotting and RNAscope technology, respectively. The results showed that the middle-aged mice had worse cognitive performance and higher Staufen expression than young mice. Embryonic inflammation induced cognitive impairment and increased Staufen expression in the middle-aged mice, whereas adolescent stress/an enriched environment would accelerated/mitigated these effects. Meanwhile, Staufen expression was closely correlated with cognitive performance. Our findings suggested embryonic inflammation can accelerate age-associated learning and memory impairments, and these effects may be related to the Staufen expression.

## Introduction

Population aging constitutes a significant public health challenge globally. Aging is associated with cognitive decline, such as spatial learning and memory impairments, which are among the earliest and most striking effects of aging (Belblidia et al., 2018). Such age-associated learning and memory impairments can have a strong and negative impact on the quality of life of the affected individuals. Consequently, it is essential to understand the normal aging process as well as the mechanisms underlying cognitive decline. However, although interest in this field has grown, the mechanisms that trigger and maintain age-related diseases remain poorly understood, and approaches to alleviate brain aging remain ineffective.

The hippocampus is an organ that is susceptible to the effects of aging, and plays an important role in spatial learning and memory (Moorthi et al., 2015; Tsai et al., 2018). There is growing evidence to suggest that hippocampal synaptic function and plasticity play a key role in age-associated learning and memory impairments (Mendelsohn and Larrick, 2012). The localization of mRNAs to synapses and the subsequent regulation of local translation has been proposed as one mechanism underlying the regulation of synaptic plasticity and establishment of hippocampus-dependent learning and memory (Ule and Darnell, 2006). However, little is known about the *in vivo* role of RNA-binding proteins (RBPs) in RNA transport to the synapse and subsequent local protein synthesis.

Staufen, a double-stranded RBP composed of five double-stranded RNA (dsRNA)-binding domains, was initially identified in a genetic screen for maternal-effect mutants in Drosophila (Bonnet-Magnaval et al., 2016). This protein is present in neurons, where it localizes to ribonucleoprotein particles in the cell body and dendrites, and has been shown to play crucial roles in the localization, translation, and stabilization of dendritic mRNA (Goetze et al., 2006). Staufen typically interacts with specific regulatory elements on the 3′ untranslated region (UTR) of mRNAs to enable their localization or regulation; these complexes then assemble into larger RNA granules, which are transported along cytoskeletal tracks by motor proteins (Sudhakaran and Ramaswami, 2017). Data reported for Drosophila, Aplysia, and the mouse all indicate that Staufen makes a crucial contribution to dendrite development, synaptic plasticity, learning, and memory (Dubnau et al., 2003; Heraud-Farlow and Kiebler, 2014; Berger et al., 2017). A recent study also found that Staufen overabundance can contribute to aberrant translation, ribostasis, and proteostasis (Gandelman et al., 2020). Collectively, these observations highlight the importance of Staufen-mediated posttranscriptional regulation in cognition.

Recent evidence has demonstrated that early exposure to adverse factors, including environmental, genetic, or a combination of both, can exacerbate age-associated cognitive impairment (Zhang et al., 2020). This view has been encompassed in the “fetal origin of adult disease” hypothesis (Benarroch, 2013). Studies have indicated that exposure to inflammation in the embryonic period (Qin et al., 2007; Abareshi et al., 2016) or to stress in adolescence (Speisman et al., 2013; Shields et al., 2017) may be involved in the occurrence of the age-associated learning and memory impairments.

Inflammation is a commonly occurring adverse factor in early life. Several studies have reported that a close relationship exists between neuroinflammation and neuropsychiatric disorders such as memory impairment, depression, and anxiety (Jayaraj et al., 2017). Administration of lipopolysaccharide (LPS), a toxic component found in the cell walls of Gram-negative bacteria, is a well-characterized and widely used model of inflammation (Boksa, 2010). Mimicking intrauterine infection and inflammation through maternal LPS exposure during pregnancy can lead not only to fetal death, growth restriction, skeletal development retardation, and preterm labor, but also to a significant increase in the synthesis and release of specific proinflammatory cytokines, such as tumor necrosis factor, interleukin-1 beta, and interleukin-6, into maternal serum, as well as markedly impair the cognitive abilities and social-behavioral performance of offspring (Wang H. et al., 2010; Badshah et al., 2016; Zhan, 2017). We previously also found that embryonic exposure to LPS-induced inflammation led to age-related spatial learning and memory impairment and corresponding neurobiochemical changes (Li X. Y. et al., 2016; Wu Z. X. et al., 2019).

Whether exposure to stress during adolescence can influence inflammation-induced cognitive impairment is not known. Studies have revealed that exposure to adolescent stress can increase the risk of disease, such as that associated with cardiovascular and metabolic disorders (Nagaraja et al., 2016). Recent research has also shown that exposure to adolescent stress results in structural and functional alterations in the developing hippocampus, including reduced long-term potentiation (LTP), and these alterations are thought to be associated with impaired spatial learning and memory (Fujioka et al., 2006). In contrast, exposure to an enriched environment (EE) in adolescence can help prevent these effects. EEs are known to provide multisensory stimulation, which induces brain plasticity following exposure to different types of objects such as toys, tunnels, ladders, and running wheels, among others, in a spacious environment (Bhagya et al., 2017). Increasing evidence suggests that an EE can exert significant ameliorative effects on neurogenesis, synaptogenesis, and learning and memory abilities (Jayaraj et al., 2017; Ferioli et al., 2019; Wu C. et al., 2019). However, it remains unclear whether an EE can counteract, at least partially, the harmful effects of embryonic inflammation on age-associated learning and memory impairments.

In brief, growing evidence has suggested that exposure to embryonic inflammation can impair spatial learning and memory in the later life; however, whether the stress/an EE in adolescence can accelerate/mitigate the age-associated cognitive impairment resulting from the embryonic inflammation remains unknown, as do the potential associated mechanisms. We speculated that the Staufen protein may be involved in these impairments caused by embryonic inflammation. In this study, we first explored whether embryonic inflammation could accelerate age-associated cognitive impairment. Subsequently, we investigated whether Staufen expression changed with age and under different treatments. Finally, we examined whether changes in Staufen expression are correlated with deficits in spatial learning and memory.

## Materials and Methods

### Animals and Drugs

**FIGURE 1:**
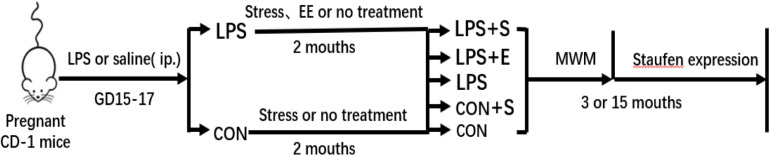
Timeline of the experiment. Pregnant mice received a daily intraperitoneal injection of LPS or normal saline during gestation days 15–17. At 2 months of age, the offspring were exposed either to a stressed or an enriched environment, following which they were randomly assigned to five groups (LPS+S, LPS, LPS+F, CON+S, and CON). A Morris water test was applied to 3-month-old and 15-month-old mice. The mice were then sacrificed for subsequent Staufen expression analysis. CON, untreated control group; LPS, lipopolysaccharide treatment group; S, group of mice exposed to stress; E, group of mice exposed to an enriched environment.

CD-1 mice (8 weeks old, 10 males and 20 females) were obtained from Hunan SJA Laboratory Animal Co., Ltd. (NO. 43004700010146; Hunan, China). The animals were maintained at a constant temperature of 22–25°C with 55 ± 5% humidity on a 12-h light–dark cycle (lights on at 07:00). Food and water were available *ad libitum*. After 2 weeks of acclimatization, female mice were paired with males at a 2:1 ratio. The presence of a vaginal plug was designated as gestational day (GD) 0. Based on our previous study (Wu Z. X. et al., 2019), during GDs 15–17, the mice received a daily intraperitoneal injection of lipopolysaccharides (LPS, 50 μg/kg) or the same volume of normal saline. To avoid stress, the offspring were only separated from their mothers at postnatal day 21, following which they were housed in polypropylene cages, 4–5 mice per cage. At 2 months of age, the offspring had exposed to a stress (S), an enriched environment (E), or an unchanged environment (CON), then assigned to five groups (LPS+S, LPS+E, LPS, CON+S, and CON, respectively). Three-month-old (3M; young) and 15-month-old (15M; aged) CD-1 mice (except those with movement disorders, hair loss, or visible tumors) were used to complete the tests described in sections “Morris Water Maze,” “Tissue Preparation,” “Immunohistochemistry,” and “Western Blotting.” The timeline of the experiment is shown in Figure 1. All animal experiments were performed in compliance with the guidelines established by the National Institutes of Health (NIH) Guide for the Care and Use of Laboratory Animals. The protocol was approved by the Center for Laboratory Animal Sciences at Anhui Medical University.

### Stress

Young (2 months old) mice in the LPS+S and CON+S were exposed to a variable sequence of chronic, mild, unpredictable stressors (Sun et al., 2018). One of the following four stressors was randomly applied per day over the 4-day cycle (4 days constituted one cycle, and the experiment lasted for seven cycles): (1) Restraint: mice were placed in a net pocket made of soft wire (35 × 40 cm^2^) to restrict their movement. Restraining time lasted for 30 min on the first day, and was extended by 10 min each day, all the while maintaining normal respiration. (2) Suspension: the mice were suspended by the tail from a crossbar (1.2 m high) for 30 min on the first day, and the duration was extended by 10 min each time, thereby sequentially increasing the stress intensity. (3) Illumination: in the normal light–dark cycle, repeated cycles of 30 min lights on/30 min lights off were applied from 19:00 to 7: 00. (4) Fasting: the food was removed between 19:00 and 7: 00, while drinking water was provided *ad libitum*.

### Enriched Environment

Mice in the LPS+E were reared in large cages to promote social and group activities. Different types of toys, such as pipes, plastic running wheels, and balls, were placed in the cage every week to increase the ability of the mice to avoid difficulties, their adaptation to the novel environment, and the amount of physical exercise until the behavioral examination was completed (Van Loo et al., 2004).

### Morris Water Maze

The Morris water maze (MWM) was used to evaluate spatial learning and memory ability (Vorhees and Williams, 2006; Tong et al., 2015; Jafarian et al., 2019). The apparatus consisted of a circular black tank (100 cm in diameter, 30 cm in height) and an escape platform (10 cm in diameter, 24 cm in height); the tank was filled with water (20–22°C) to a depth of 25 cm. To guide spatial navigation, the periphery of the tank was surrounded by a white curtain with three black conspicuous markers (a square, a circle, and a triangle). The test was performed four times daily, with 15-min intervals between trials, and lasted for 7 days. On day 1, the mice were placed on the escape platform for 30 s before the first trial began. Then, the mice were placed in the water facing the pool wall and allowed to swim freely for 60 s to find the escape platform (acquisition trials). If they failed to find the platform within 60 s, they were gently guided to the platform before being removed. At the end of each test, regardless of whether or not they found the platform, the mice were allowed to rest on the platform for 30 s and then put back in their home cage to keep warm. The position of the platform remained constant throughout training, whereas the starting points were randomly selected. The probe test (removal of the platform for 60 s) was performed 2 h after the last acquisition trial on the last day of the test. The time it takes for mice to reach the escape platform (escape latency) is the most commonly used measure of learning performance; however, escape latency can be affected by swimming speed, which usually declines with age (van der Staay and de Jonge, 1993). Therefore, in this study, the total swimming distance in the learning phase was used as the spatial learning ability and the percent distance swam in the target quadrant in the probe test was used as the memory performance (Chen et al., 2015; Zhang et al., 2020). ANY-maze software (Stöelting, United States) was used to record the distance swam.

### Tissue Preparation

Six mice per group were anesthetized with chloral hydrate (360 mg/kg, i.p.), and sacrificed by cervical dislocation. The brains were then rapidly removed from the skull and cut along the midsagittal plane on ice. The left hippocampus was stored at -80°C for western blot analysis. The right hemisphere was fixed in 4% paraformaldehyde at 4°C for 3 days, and then paraffin-embedded into blocks. Continuous coronal sections (3 μm) were prepared using a Leica Microtome (Leica RM 2135, Germany) for subsequent immunohistochemistry and RNAscope assays.

### Immunohistochemistry

The streptavidin–biotin–peroxidase complex (SABC) method was used for immunohistochemical staining, as previously described (Nagashima et al., 1992). After conventional dewaxing and hydration, the sections were treated with periodate-inactivated enzyme for 1 min to deactivate endogenous peroxidases and microwaved in citrate buffer (0.01 mol/L, pH 6.0) for 20 min for antigen retrieval. Then, the sections were treated with 5% fetal bovine serum albumin in phosphate-buffered saline at 37°C for 10 min to minimize non-specific staining. The tissue sections were treated with an anti-Staufen antibody (1:200; ab73478, Abcam, United States) and incubated overnight at 4°C. The next day, the sections were rewarmed at 37°C for 40 min, incubated with a secondary antibody (biotin-labeled goat anti-rabbit IgG), and subsequently treated with SABC (SA1022; Wuhan Boster Bioengineering Co., Wuhan, China) at 37°C for 20 min. Finally, staining was visualized using diaminobenzidine (ZLI-9018; ZsBio, Beijing, China). Images of the whole hippocampus (4 × 10) and the three subregions [cornu ammonis (CA)1, CA3, and dentate gyrus (DG); 20 × 10] were obtained using a digital scanner (Panoramic MIDI).

### Western Blotting

Western blotting was performed as previously reported (Wu T. et al., 2019; Shen et al., 2020). Hippocampal tissue was lysed in RIPA lysis buffer, and protein concentrations were determined using the bicinchoninic acid method. Protein samples were separated by 15% SDS–PAGE and then transferred onto PVDF immunoblotting membranes. The membranes were blocked with 5% dry milk containing 0.1% Tween 20 for 2 h and incubated with a primary monoclonal antibody against Staufen (1:1000; ab73478, Abcam) at 4°C overnight. The membranes were then incubated with horseradish peroxidase-conjugated secondary antibodies (1:10,000; ZB2301, ZsBio) for 2 h at room temperature followed by chemiluminescence detection. Immunoreactive bands at 63 kDa (Staufen) and 43 kDa (beta-actin, internal standard) denoted positive expression. Densitometric quantification of the band intensities was performed using Image-J. The ratio of the optical density of the anti-Staufen antibody to that of the anti-beta-actin antibody in each sample was calculated as the relative Staufen protein level.

### RNAscope *in situ* Hybridization Assay

RNAscope *in situ* hybridization was performed as previously described (Domi et al., 2019; Gavini et al., 2020). The RNAscope assay was performed on formalin-fixed, paraffin-embedded (FFPE) tissue. Briefly, tissue sections (3 μm) were deparaffinized in xylene, rehydrated in an ethanol series, and then incubated with 5–8 drops of H_2_O_2_ for 10 min. The sections were incubated in citrate buffer (10 nmol/L, pH 6.0) at 100°C for 15 min. An ImmEdge pen was used to create a barrier around each section. The slides were incubated for 30 min at 40°C with the RNAscope protease plus reagent, and then with the Staufen mRNA target probes (ACD, 322381) for 2 h at 40°C in the HybEZ hybridization oven. The sections were then serially incubated with four amplifier probes (30 min each for steps 1 and 2, 15 min each for steps 3 and 4) at 40°C. After removing excess liquid, the TSAplus fluorescent dye was added to the slide for 30 min at 40°C, and then the RNAscope multichannel fluorescent second-generation HRP blocker was also added to the slide for 15 min at 40°C. Finally, the sections were counterstained with DAPI to visualize the nuclei. The slides were cover-slipped, air dried, and stored at 4°C. Fluorescent signals from RNAscope probe hybridization were examined on a laser-scanning confocal microscope (×40 objective; Zeiss LSM 700). To visualize the entire brain section, tile scan images were obtained using an Olympus IX71 fluorescence microscope (Olympus, Tokyo, Japan) equipped with a PXL37 CCD camera (Photometrics, Tucson, AZ, United States). Fluorescence images were semi quantitatively analyzed in ImageJ. The number of dots in the Staufen mRNA-positive cells relative to the negative control was calculated as the relative levels of Staufen mRNA. The negative control was an internal standard and was used to set the light source and exposure time of image acquisition to acceptable background levels.

### Statistical Analysis

The sample size was calculated by G^∗^Power software (ver. 3.1.7, Franz Faul, Universitat Kiel, Germany) The α error was set at 0.05 and power (1-β) at 0.8 and the essential total sample size for each group in the behavioral assessments and molecular experiments was calculated as 6–8. Parametric data were expressed as means ± standard error of the mean (SEM). For the learning performance in the MWM, repeated-measures analysis of variance (rm-ANOVA) was used to analyze the learning data, with day, age, and group as independent variables. The memory percentage of distance from the MWM test and data from the western blotting and RNAscope assays were evaluated by one-way ANOVA with age or treatment as independent variables. Fisher’s least significant difference test was performed to compare the differences among the groups. The correlations between the MWM performance and the relative levels of Staufen protein and mRNA in the hippocampus were analyzed using Pearson’s correlation coefficient. The Statistical Package for Social Sciences (SPSS, version 20.0) was used for analyses, and significance was assumed at *P* < 0.05.

## Results

### Performances in the MWM

#### Learning Phase

**FIGURE 2:**
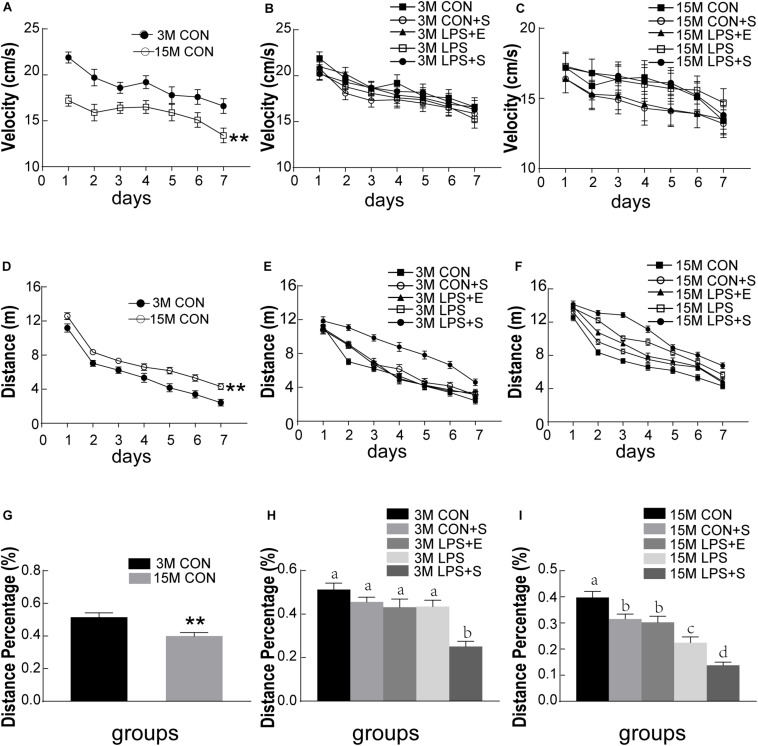
Morris water maze performance of CD-1 mice under different treatments. The swimming velocity in the learning phase is shown in **(A–C)**; the swimming distances in the learning phase is shown in **(D–F)**; and the memory percentage of distance in the target quadrant is shown in **(G–I)**. The age effects are depicted in **(A,D,G)** and the treatment effects in **(B,E,H)** for the 3-month-old mice (3M) mice and **(C,F,I)** for 15-month-old (15M) mice. All values are presented as means ± SEM (*n* = 8 male mice/group). **P < 0.01. Different lowercase letters (a/b/c) denote significant differences, and a > b > c > d. CON, untreated control group; LPS, lipopolysaccharide treatment group; S, group of mice exposed to stress; E, group of mice exposed to an enriched environment.

The swimming velocity was significantly lower in the 15M mice than in the 3M mice in the CON [*F_(__1_,_14__)_* = 16.459, *P* < 0.01], suggesting an age-related decline in motor ability in middle-aged mice (Figure 2A). LPS, stress and EE did not significantly affect the swimming velocity of either the 3M and 15M mice (*Ps* > 0.05; Figures 2B,C). Thus, the swimming distance was analyzed as an indicator of learning ability. The distance swam decreased progressively and daily for all the mice combined [*F_(__6_,_84__)_* = 101.993, *P* < 0.01]. Furthermore, the 15M mice swam greater distances than the 3M mice both in the CON [*F_(__1_,_14__)_* = 26.595, *P* < 0.01; Figure 2D] and in the other treatment groups (CON+S, LPS, LPS+E, and LPS+S) (*Ps* < 0.01; Supplementary Figures S1A–D). There were significant differences in swimming distance among the treatment groups in both the 3M mice [*F_(__4_,_35__)_* = 18.344, *P* < 0.01, Figure 2E] and 15M mice [*F_(__4_,_35__)_* = 46.755, *P* < 0.01; Figure 2F]. Irrespective of age, mice in the LPS+S swam significantly longer distances than those in the other four groups (*Ps* < 0.01). In addition, for the 15M mice, the CON exhibited the shortest swimming distances among the five groups (*Ps* < 0.01). Moreover, the LPS swam significantly longer distances than those in the LPS+E and CON-S (*Ps* < 0.01); however, no significant difference in the distance was observed between the latter two groups (*P* > 0.05). The effect of interaction of ages × days on swimming distance was not significant [*F_(__6_,_84__)_* = 0.459, *P* > 0.05].

#### Memory Phase

The percentage of distance traversed in the target quadrant was significantly lower in 15M mice than in 3M mice in both the CON (*t* = 3.057; *P* < 0.01; Figure 2G) and the other treatment groups (CON+S, LPS, LPS+E, and LPS+S) (*Ps* < 0.05; Supplementary Figures S2A–D). There were significant differences in the percentage of distance among the five groups for both 3M [*F_(__4_,_39__)_* = 11.522, *P* < 0.01; Figure 2H] and 15M mice [*F_(__4_,_39__)_* = 23.253, *P* < 0.01; Figure 2I]. Irrespective of age, mice in the LPS+S exhibited a significantly lower percentage of distance than those in the other four groups (*Ps* < 0.01). In addition, at 15 months of age, mice in the CON showed a significantly greater percentage of distance than those in the other four groups (*Ps* < 0.01); meanwhile the percentage of distance was significantly lower in the LPS than in the CON+S and LPS+E (*Ps* < 0.05); no significant difference was observed between the latter two groups (*P* > 0.05).

### Staufen Expression in the Hippocampus

#### Protein Level

**FIGURE 3:**
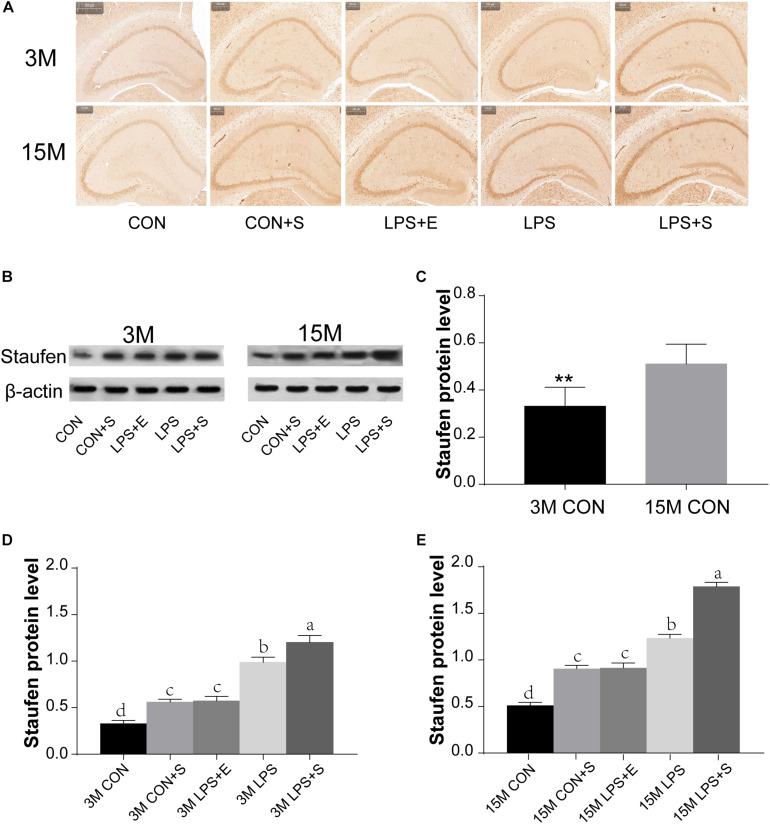
The hippocampal Staufen protein level in CD-1 mice under different treatments. **(A)** Representative images of Staufen immunohistochemical staining. **(B)** Western blotting analysis of Staufen levels; beta-actin was used as a loading control. **(C)** Staufen protein levels in the CON at different ages. **(D,E)** Hippocampal Staufen protein levels in 3-month-old (3M) and 15-month-old (15M) mice in the different treatment groups. Scale bar = 200 μm. All values are presented as means ± SEM (*n* = 6 male mice/group). **P < 0.01. Different lowercase letters (a/b/c) denote significant differences, and a > b > c > d. CON, untreated control group; LPS, lipopolysaccharide treatment group; S, group of mice exposed to stress; E, group of mice exposed to an enriched environment.

The level of Staufen protein in the hippocampus was measured by immunohistochemistry and western blotting. Staufen protein staining was mainly localized to the cellular layer of the hippocampus, moreover, the immunoreactivity was more evident in 15M mice than in 3M mice, and greater in the LPS+S than the CON (Figures 3A,B and Supplementary Figures S3A,B). The 15M mice had a significantly higher Staufen protein level than the 3M mice in both the CON (*t* = 3.753; *P* < 0.01; Figure 3C) and the other treatment groups (CON+S, LPS, LPS+E, and LPS+S) (*Ps* < 0.01; Supplementary Figures S4A–D). There were significant differences in hippocampal Staufen protein levels among the five groups for both 3M [*F_(__4_,_29__)_* = 47.516, *P* < 0.01, Figure 3D] and 15M mice [*F_(__4_,_29__)_* = 127.422, *P* < 0.01, Figure 3E]. Regardless of age, the LPS+S had the highest, and the CON the lowest, Staufen protein levels (*Ps* < 0.05); moreover, Staufen protein levels were significantly higher in the LPS than in the CON+S and LPS+E (*Ps* < 0.05); but no significantly differences was found between the latter two groups (*Ps* > 0.05).

#### mRNA Level

**FIGURE 4:**
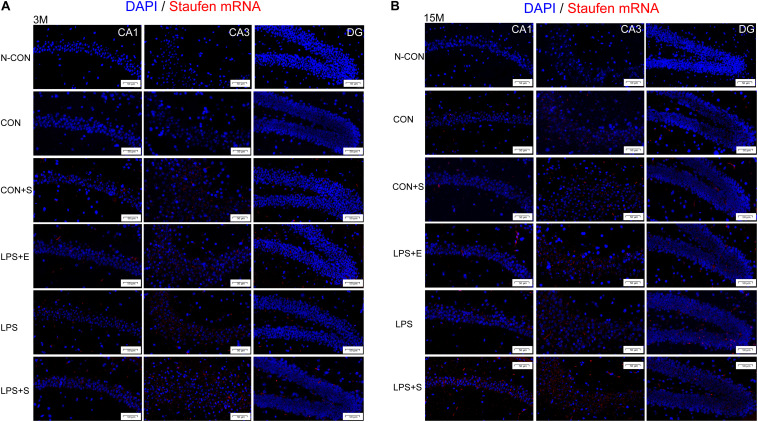
Representative photomicrographs of Staufen mRNA levels relative to the negative control in the different hippocampal subregions of CD-1 mice of different ages exposed to different treatments. The results for the different hippocampal subregions in 3-month and 15-month-old mice are shown in **(A,B)**, respectively. The red staining represents a positive Staufen mRNA staining. Scale bar = 50 μm. CA, cornu ammonis; DG, dentate gyrus; N-CON, the negative control group; CON, untreated control group; LPS, lipopolysaccharide treatment group; S, group of mice exposed to stress; E, group of mice exposed to an enriched environment.

**FIGURE 5:**
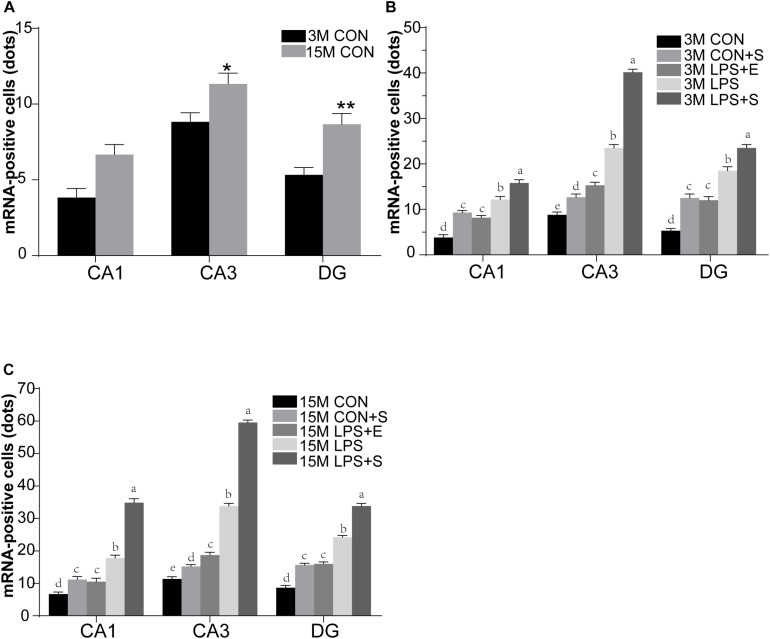
The relative levels of Staufen mRNA in the different hippocampal subregions of CD-1 mice of differing ages exposed to different treatments. The age effect is shown in **(A)** and the treatment effects for 3-month-old **(B)** and 15-month-old **(C)** mice. All values are presented as means ± SEM (*n* = 6 male mice/group). *P < 0.05, **P < 0.01. Different lowercase letters (a/b/c) denote significant differences, and a > b > c > d. CON, untreated control group; LPS, lipopolysaccharide treatment group; S, group of mice exposed to stress; E, group of mice exposed to an enriched environment.

Staufen mRNA expression was mainly detected in the cell layer in each hippocampal subregion (CA1, CA3, and DG) (Figures 4A,B). The 15M mice had significantly higher Staufen mRNA levels in the corresponding hippocampal subregions in the CON (CA1, *t* = 3.157, *P* = 0.010; CA3, *t* = 2.677, *P* = 0.023; DG, *t* = 3.835, *P* = 0.003. Figure 5A) than the 3M mice; a similar tendency was observed for mice in the other treatment groups (*Ps* < 0.05; Supplementary Figure S5A–D). A significant effect of treatment on Staufen mRNA levels was found among the five groups for both 3M and 15M mice in the CA1 [*F_(__4_,_29__)_* = 57.294, *P* < 0.01; *F_(__4_,_29__)_* = 132.288, *P* < 0.01], CA3 [*F_(__4_,_29__)_* = 323.219, *P* < 0.01; *F_(__4_,_29__)_* = 679.549, *P* < 0.01], and DG [*F_(__4_,_29__)_* = 78.072, *P* < 0.01; *F_(__4_,_29__)_* = 223.429, *P* < 0.01] subregions. Regardless of age, Staufen mRNA levels in the CA1, CA3, and DG (*Ps* < 0.01) subregions presented significantly higher levers in the LPS+S, followed by the LPS, with the CON showing the lowest Staufen mRNA levels. Furthermore, Staufen mRNA levels in the CA3 subregion were significantly higher in the LPS+E than in the CON+S (*Ps* > 0.05; Figures 5B,C).

### Correlations Between Cognitive Performance and Staufen Expression

#### Correlations Between Cognitive Performance and Staufen Protein Levels

Table 1 depicts the correlation between the MWM performances and hippocampal Staufen protein levels. Staufen protein levels were positively correlated with the learning swimming distance (*Ps* < 0.01) and negatively correlated with the memory percentage of distance (*Ps* < 0.05) in all the mice combined. When the groups were separated, Staufen protein levels in the 3M LPS+S and 15M LPS+S, LPS, and LPS+E were positively correlated with the learning swimming distance (*Ps* < 0.05). Furthermore, Staufen protein levels in the 3M LPS+S and 15M LPS+S, LPS were negatively correlated with the memory percentage of distance (*Ps* < 0.05). Supplementary Figure S6A shows scatter plots of the group that had significantly correlation coefficients in the Table 1.

**TABLE 1:** The correlations between hippocampal Staufen protein levels and learning and memory performance.

**Cognitive phases**	**Age**	**Group**	**Staufen protein levels**
			***r (p)***
Swimming	3 months	All mice	0.729 (0.000)**
distance		CON	0.777 (0.069)
		CON+S	0.619 (0.191)
		LPS+E	0.213 (0.685)
		LPS	0.353 (0.493)
		LPS+S	0.964 (0.002)**
	15 months	All mice	0.940 (0.000)**
		CON	0.710 (0.114)
		CON+S	0.605 (0.203)
		LPS+E	0.888 (0.018)*
		LPS	0.874 (0.023)*
		LPS+S	0.880 (0.021)*
Percent	3 months	All mice	−0.699 (0.000)**
distance swam		CON	−0.417 (0.411)
in the target		CON+S	−0.295 (0.571)
quadrant		LPS+E	−0.229 (0.662)
		LPS	−0.413 (0.416)
		LPS+S	−0.890 (0.017)**
	15 months	All mice	−0.893 (0.000)**
		CON	−0.379 (0.459)
		CON+S	−0.480 (0.335)
		LPS+E	−0.685 (0.133)
		LPS	−0.955 (0.003)**
		LPS+S	−0.953 (0.003)**

*Denotes significant correlation coefficients (*P < 0.05, **P < 0.01). CON, untreated control group; LPS, lipopolysaccharide treatment group; S, group of mice exposed to stress; E, group of mice exposed to an enriched environment.

#### Correlations Between Performance and Staufen mRNA Levels

Table 2 depicts the correlations between the MWM performances and hippocampal Staufen mRNA levels. For all the mice combined, Staufen mRNA levels in all the hippocampal subregions were positively correlated with the learning swimming distance and negatively correlated with the memory percentage of distance (*Ps* < 0.05). Significantly positive correlations were found between learning swimming distance and Staufen mRNA levels in the CA1 and CA3 subregions of 3M LPS+S and 15M LPS+S, LPS, LPS+E, and CON+S mice (*Ps* < 0.05); and the DG subregion of 3M LPS+S and 15M LPS+S, LPS (*Ps* < 0.05). However, negatively correlations were found between the Staufen mRNA levels and the memory percentage of distance in the CA1 subregion of 3M LPS+S and 15M LPS+S, LPS, and CON+S mice (*Ps* < 0.05); the CA3 subregion of 3M LPS+S and 15M LPS+S and LPS mice (*Ps* < 0.05); and the DG subregion of 15 LPS+S and LPS mice (*Ps* < 0.05). Supplementary Figures S6B,C shows scatter plots of the group that had significantly correlation coefficients in the Table 2.

**TABLE 2:** The correlations between hippocampal Staufen mRNA levels and learning and memory performance.

**Cognitive phase**	**Age**	**Group**	**Staufen mRNA levels**		
			**CA1**	**CA3**	**DG**
			***r (p)***	***r (p)***	***r (p)***
Learning swimming	3 months	All mice	0.769 (0.000)**	0.780 (0.000)**	0.739 (0.000)**
distance		CON	0.773 (0.071)	0.290 (0.577)	0.355 (0.490)
		CON+S	0.605 (0.204)	0.539 (0.269)	0.751 (0.086)
		LPS+E	0.590 (0.217)	0.612 (0.197)	0.192 (0.715)
		LPS	0.225 (0.669)	0.315 (0.543)	0.752 (0.084)
		LPS+S	0.841 (0.036)*	0.824 (0.044)*	0.820 (0.046)*
	15 months	All mice	0.901 (0.000)**	0.894 (0.000)**	0.930 (0.000)**
		CON	0.403 (0.428)	0.611 (0.198)	0.693 (0.127)
		CON+S	0.880 (0.021)*	0.820 (0.046)*	0.113 (0.831)
		LPS+E	0.897 (0.015)*	0.945 (0.004)**	0.585 (0.222)
		LPS	0.931 (0.007)**	0.935 (0.006)**	0.876 (0.022)*
		LPS+S	0.904 (0.013) *	0.903 (0.014) *	0.881 (0.020)*
Percent distance swam	3 months	All mice	−0.739 (0.000)**	−0.725 (0.000)**	−0.675 (0.000)**
in the target quadrant		CON	−0.425 (0.401)	−0.566 (0.242)	−0.265 (0.612)
		CON+S	−0.138 (0.794)	−0.574 (0.234)	−0.325 (0.530)
		LPS+E	−0.229 (0.662)	−0.161 (0.760)	−0.018 (0.973)
		LPS	−0.555 (0.253)	−0.351 (0.495)	−0.124 (0.815)
		LPS+S	−0.938 (0.006)**	−0.825 (0.043)*	−0.791 (0.061)
	15 months	All mice	−0.861 (0.000)**	−0.839 (0.000)**	−0.885 (0.000)**
		CON	−0.476 (0.340)	−0.026 (0.962)	−0.715 (0.111)
		CON+S	−0.825 (0.043)*	−0.794 (0.059)	−0.072 (0.892)
		LPS+E	−0.542 (0.267)	−0.788 (0.063)	−0.651 (0.161)
		LPS	−0.884 (0.019)*	−0.833 (0.039)*	−0.842 (0.035)*
		LPS+S	−0.922 (0.009)**	−0.887 (0.018)*	−0.871 (0.024)*

*Denotes significant correlation coefficients (*P < 0.05, **P < 0.01). CA, cornu ammonis; DG, dentate gyrus; CON, untreated control group; LPS, lipopolysaccharide treatment group; S, group of mice exposed to stress; E, group of mice exposed to an enriched environment.

## Discussion

Early life is a critical developmental period, and experiences in this stage, i.e., embryonic stage and adolescence have been shown to have a long-term influence on developmental and aging processes (Li X. Y. et al., 2016; Höltge et al., 2019). The embryonic period is associated with a greater vulnerability to bacterial and viral infections owing to an immature immune system (Khan et al., 2017; Alsaif et al., 2018). It is well documented that pregnant animals have increased sensitivity to LPS than non-pregnant ones (Kunnen et al., 2014). In this study we found that the embryonic exposure to LPS could accelerate the cognitive impairments in middle-aged mice, and adolescent stress/an EE could exacerbate/relieve this effect. Therefore, avoiding embryonic infection was of great significance to guide healthy borning and fine rearing, and avoiding stress and enriching the environment in the adolescence could further mitigate cognitive impairment. Moreover, we also demonstrated that increased Staufen expression in the hippocampus, was correlated with the impaired cognition in the different treatment groups. To some extent, our results provide new insights into the mechanisms involved in cognitive decline resulting from embryonic inflammation.

### The Effects of Embryonic Inflammation and Adolescent Psychosocial Environment (Stress or EE) on Cognition in Middle-Aged Mice

In the current study, the 15M CON showed a significantly lower swimming velocity than the 3M CON, suggesting a decline in motor ability in middle-aged mice, which is consistent with the previous results (Chen et al., 2015; Zhang et al., 2020; Duan et al., 2020). van der Staay and de Jonge (1993) showed that if the learning ability of two mice was equal, the slower mice may need more swimming time to reach the escape platform (escape latency) than the faster mice; however, the swimming distance had no significantly difference. Consequently, we assessed the swimming distance as an indicator of spatial learning and memory in our study.

Our results showed that spatial learning and memory were impaired in the middle-aged mice. This result was consistent with those of previous studies that indicated that age-related decline in cognitive impairment begins at 12–13 months of age in this strain mouse (Wang H. et al., 2010; Wang et al., 2020). Several studies have suggested that embryonic inflammation and adolescent stress can accelerate brain age-associate cognitive impairment (Li X. W. et al., 2016; Bhagya et al., 2017; Wang et al., 2020). In contrast, an adolescent EE can relieve the deficits in hippocampal synaptic plasticity, memory, and anxiety caused by chronic stress (Bhagya et al., 2017). Our results indicated that embryonic inflammation or adolescent stress alone does not affect spatial learning and memory in young adults; however, together, these two factors can have a synergistic, deteriorative effect on cognitive behavior. Additionally, the current results also suggested that embryonic inflammation can impair spatial learning and memory abilities in the middle-aged mice to a greater extent than the effect of adolescent stress. Furthermore, we did not observe any difference between the LPS+E and CON+S, suggesting that an EE in adolescence can mitigate the age-associated cognitive impairment resulting from embryonic inflammation.

### The Effects of Embryonic Inflammation and Adolescent Psychosocial Environment (Stress or EE) on Staufen Expression in Middle-Aged Mice

Staufen, a dsRBP, was initially identified in a screen for anterior–posterior patterning mutants in Drosophila embryos. Staufen is recruited to stress granules during the stress response, and can play an important role in mRNA localization, translation, and/or stability (Lebeau et al., 2008; Zimyanin et al., 2008; Paul et al., 2018). To date, no study has investigated whether aging and embryonic inflammation can affect Staufen expression. In the current study, for the first time, we explored the changes in Staufen expression (protein and mRNA) that occur as a result of aging and exposure to embryonic inflammation, or the effect of adolescent psychosocial environment (stress or an EE) on hippocampal Staufen expression following exposure to embryonic inflammation.

Studies have shown that Staufen protein levels are markedly upregulated in multiple cell and animal models of human neurodegenerative diseases, including those associated with mutations in presenilin 1, and microtubule-associated protein tau, as well as in stroke and myotonic dystrophy (Gandelman et al., 2020). Our results are consistent with those of studies showing that Staufen expression would increase following exposure to a variety of acute noxious stimuli (Bonnet-Magnaval et al., 2016). Here, our data indicated a significant effect of age, and this age-related increase in hippocampal Staufen expression (protein and mRNA) was in accordance with the behavioral change observed, i.e., impaired spatial learning and memory abilities. Moreover, treatment also exerted a significant effect on Staufen expression. Regardless of age, we found that the LPS+S showed the highest levels of hippocampal Staufen protein, followed by the LPS, whereas the CON had the lowest levels, however, no significant difference in the Staufen protein levels was found between the LPS+E and the CON+S. Collectively, these observations support that the more detrimental the factor, the higher the Staufen expression, indicating that the effect of embryonic inflammation was significantly stronger than the effect of adolescent stress, while both factors combined exerted the strongest effect. However, an adolescent EE could partially reverse the changes in Staufen expression, further illustrating that stress upregulated Staufen protein levels. Notably, the levels of Staufen mRNA was significantly higher in the CA3 subregion of the LPS+E than in the CON+S, indicating that an EE could partially reverse the change of Staufen mRNA expression resulting from the embryonic inflammation, and the effect was more obvious in the CA1 and DG subregions than in the CA3 subregion.

### The Association Between Altered Staufen Expression and Cognition

Several studies have indicated that cognitive impairment is associated with impaired synaptic plasticity. For example, embryonic inflammation and adolescent stress are shown to inhibit dendritic growth, induce neuronal remodeling and impaired synaptic transmission and plasticity, and lead to cognitive impairment in the aged mice (Lesuis et al., 2019; Tellez-Merlo et al., 2019; Wang et al., 2020). In contrast, an adolescent EE can partly reverse behavioral and synaptic abnormalities resulting from embryonic inflammation (Andoh et al., 2019). Under normal circumstances, Staufen is implicated in the transport and regulation of dendritic mRNA, so downregulation of Staufen expression can lead to a significant reduction in the number of dendritic spines and miniature excitatory postsynaptic currents, which are generally assumed to contribute to impaired synaptic plasticity (Goetze et al., 2006; Popper et al., 2018). However, following exposure to acute stress, Staufen protein levels increase, thereby increasing cellular sensitivity to apoptosis. Analogously, Staufen overabundance contributes to aberrant translation, ribostasis, and proteostasis (Ravanidis et al., 2018; Gandelman et al., 2020). This indicates that both knockdown and overexpression of Staufen can impair synaptic plasticity, and suggests that the relationship between Staufen expression and synaptic plasticity is complex (Timmerman et al., 2013). To date, no studies have investigated the correlation between Staufen expression and memory. Our results suggest that the cognitive impairment induced by the embryonic inflammation may be related to changes in Staufen protein levels.

Consistent with this hypothesis, our correlation analysis indicated that Staufen expression (protein and mRNA) was significantly correlated with cognitive ability in all the treatment groups. These findings provided the first evidence that Staufen expression is associated with impaired spatial learning and memory, as observed in the MWM test. Notably, this correlation was also age-dependent and treatment-related. A positive correlation was found between Staufen protein levels and the learning swimming distance, while a negative correlation was recorded between Staufen protein levels and the memory percentage of distance. These results suggested that the increase in hippocampal Staufen protein levels was associated with the observed age-associated learning and memory impairments following exposure to embryonic inflammation. Moreover, the pattern of correlation between cognitive performance and Staufen mRNA or protein levels was similar, further supporting that the changes in Staufen levels occured at the level of transcription. Specifically, a positive correlation was found between the learning swimming distance and Staufen mRNA levels in the CA1, CA3 subregions in all the treatment groups (LPS+S, LPS, LPS+E, and CON+S), and the DG subregion in the LPS+S and LPS. In contrast, a negative correlation was recorded between the memory percentage of distance and Staufen mRNA levels in the CA1, CA3, and DG subregions in the LPS+S and LPS; and in the CA1 subregion of the CON+S. These results further suggest that the impaired cognitive performance induced by exposure to embryonic inflammation may be attributable to increased Staufen transcription, which then leads to increased translation. This occurs preferentially in the CA1 subregion, followed by the CA3 subregion, and lastly in the DG subregion, an effect that was dependent on the intensity of the adverse stimulus. Meanwhile, Staufen mRNA levels might be more related to impaired learning ability.

In brief, our study is the first to report that exposure to embryonic inflammation and adolescent stress could, respectively, and accumulatively aggravate the age-associated learning and memory impairments, while an adolescent EE could ameliorate the changes resulting from embryonic inflammation. Secondly, to the best of our knowledge, this study is the first to report that age and embryonic inflammation can enhance the hippocampal Staufen expression at both the protein and mRNA levels, while adolescent stress/an EE can partially increase/reverse this effect. Thirdly, our results also indicated that the changes in hippocampal Staufen expression were closely correlated with impaired spatial learning and memory abilities, especially during “pathological” aging. This suggests that the learning and memory impairment resulting from embryonic inflammation may be related to changes in Staufen protein levels.

Our study also had several limitations. Firstly, it has recently been revealed that left–right anatomical and functional differences exist in the rodent hippocampus (Sakaguchi and Sakurai, 2020). In our study, a considerable amount of brain tissue was needed for the experiment, and because the unilateral hippocampal tissue available to us may not have been enough to meet the needs of the experiment, we did not account for these differences. Nonetheless, to ensure consistency in the experiment, all brain tissue was prepared in the same manner. Secondly, because we designed the experiment with a focus on the effects of embryonic inflammation, and the factors that would aggravate or alleviate these effects, so we did not set up an LPS+S+E group to investigate the compound effect. We will further enrich our groups in our subsequent study. Thirdly, we must admit that we can only find this phenomenon, but we didn’t invest the mechanism underlying age-associated learning and memory impairments, but we have provided a new insight into the mechanism of cognitive impairment caused by embryonic infection, and we still need to further clarify it in our future research.

## Data Availability Statement

All datasets presented in this study are included in the article/Supplementary Material.

## Ethics Statement

The animal study was reviewed and approved by the Center for Laboratory Animal Sciences at Anhui Medical University.

## Author Contributions

Y-FW conceived and designed the study, performed the experiments, and drafted the manuscript. Y-MZ and H-HG performed the experiments. C-YR and Z-ZZ performed the behavioral test and collected the data. LC and FW designed the study and performed the statistical analysis. G-HC designed the study and revised the manuscript. All authors read and approved the final manuscript.

## Conflict of Interest

The authors declare that the research was conducted in the absence of any commercial or financial relationships that could be construed as a potential conflict of interest.
