# Supplementary material for: Effects of Embryonic Inflammation and Adolescent Psychosocial Environment on Cognition and Hippocampal Staufen in Middle-Aged Mice
Source: Front Aging Neurosci. 2020 Sep 11;12:578719. doi: 10.3389/fnagi.2020.578719 (PMC7516039; doi:10.3389/fnagi.2020.578719)
Supplement: Supplementary file 1 [file Table_1.DOCX]

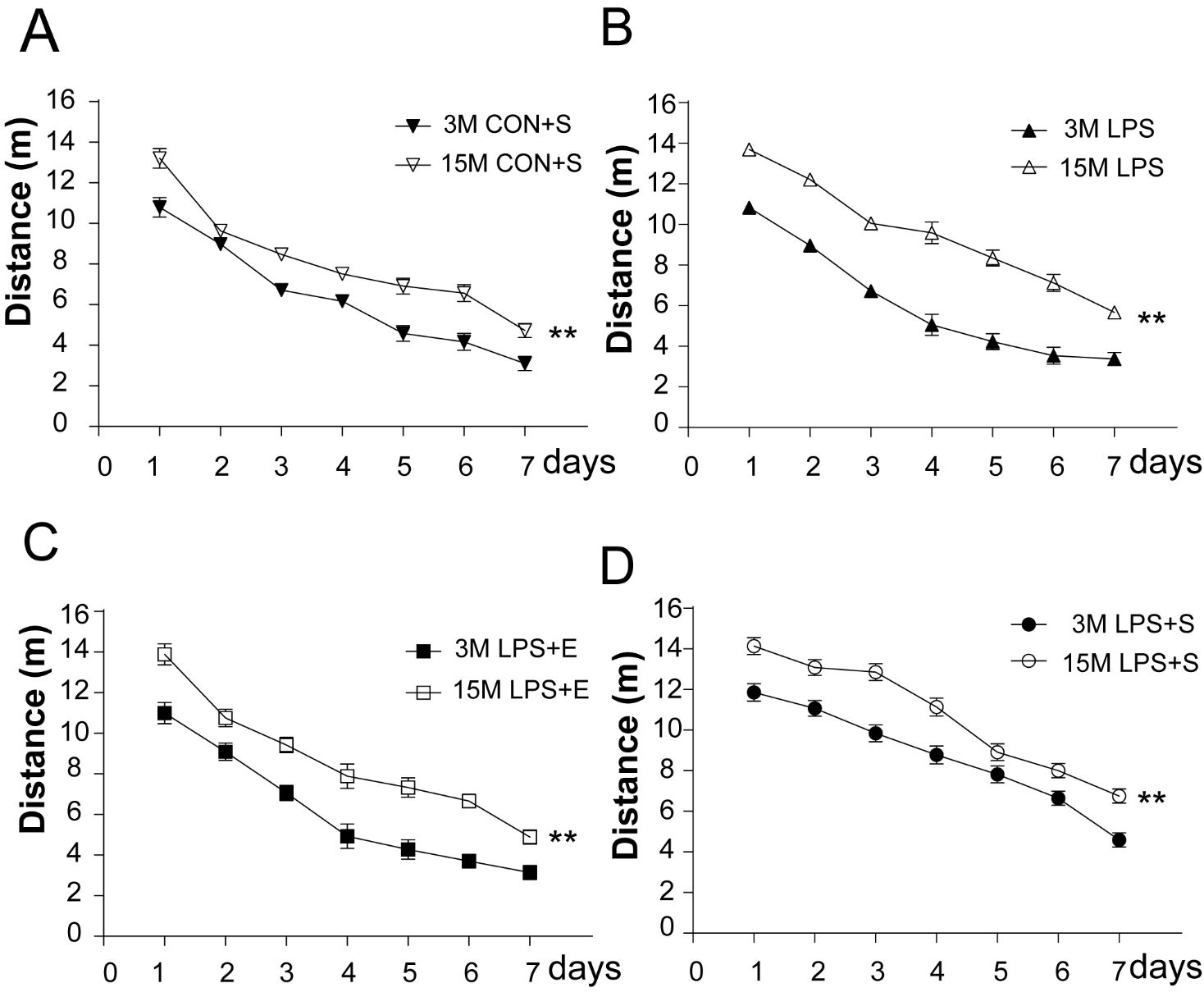


**Supplemental Figure 1.** The learning swimming distance of the Morris water maze (MWM) test for mice subjected to the different treatments (except for the CON group). The 15M mice swam longer distances than 3M mice in the CON+S (**A**), LPS (**B**), LPS+E (**C**), and LPS+S (**D**). Error bars = SEM. *N* = 8 male mice/group. **P* < 0.05, ***P* < 0.01 comparison between groups undergoing the same treatment. 3M, 3-month-old mice; 15M, 15-month-old mice; CON, untreated control group; LPS, lipopolysaccharide treatment group; S, group of mice exposed to stress; E, group of mice exposed to an enriched environment.


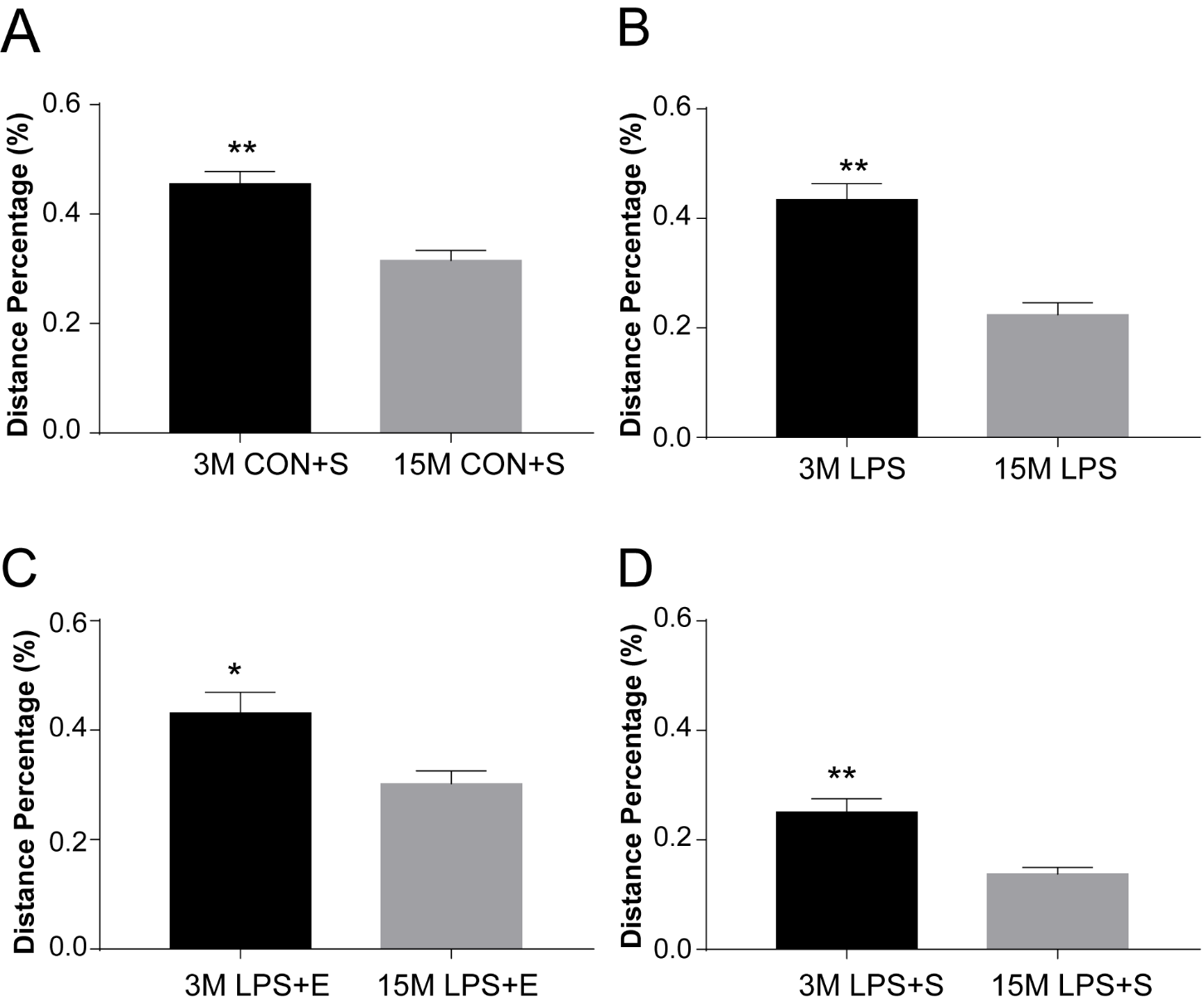


**Supplemental Figure 2.** The memory percentage of distance in the target quadrant of the Morris water maze (MWM) test for mice subjected to different treatments (except for the CON group). The 15M mice exhibited lower memory percentage of distance in the target quadrant than 3M mice in the CON+S (**A**), LPS (**B**), LPS+E (**C**), and LPS+S (**D**) groups. Error bars = SEM. *N* = 8 male mice/group. **P* < 0.05, ***P* < 0.01 comparison between groups undergoing the same treatment. 3M, 3-month-old mice; 15M, 15-month-old mice; CON, untreated control group; LPS, lipopolysaccharide treatment group; S, group of mice exposed to stress; E, group of mice exposed to an enriched environment.


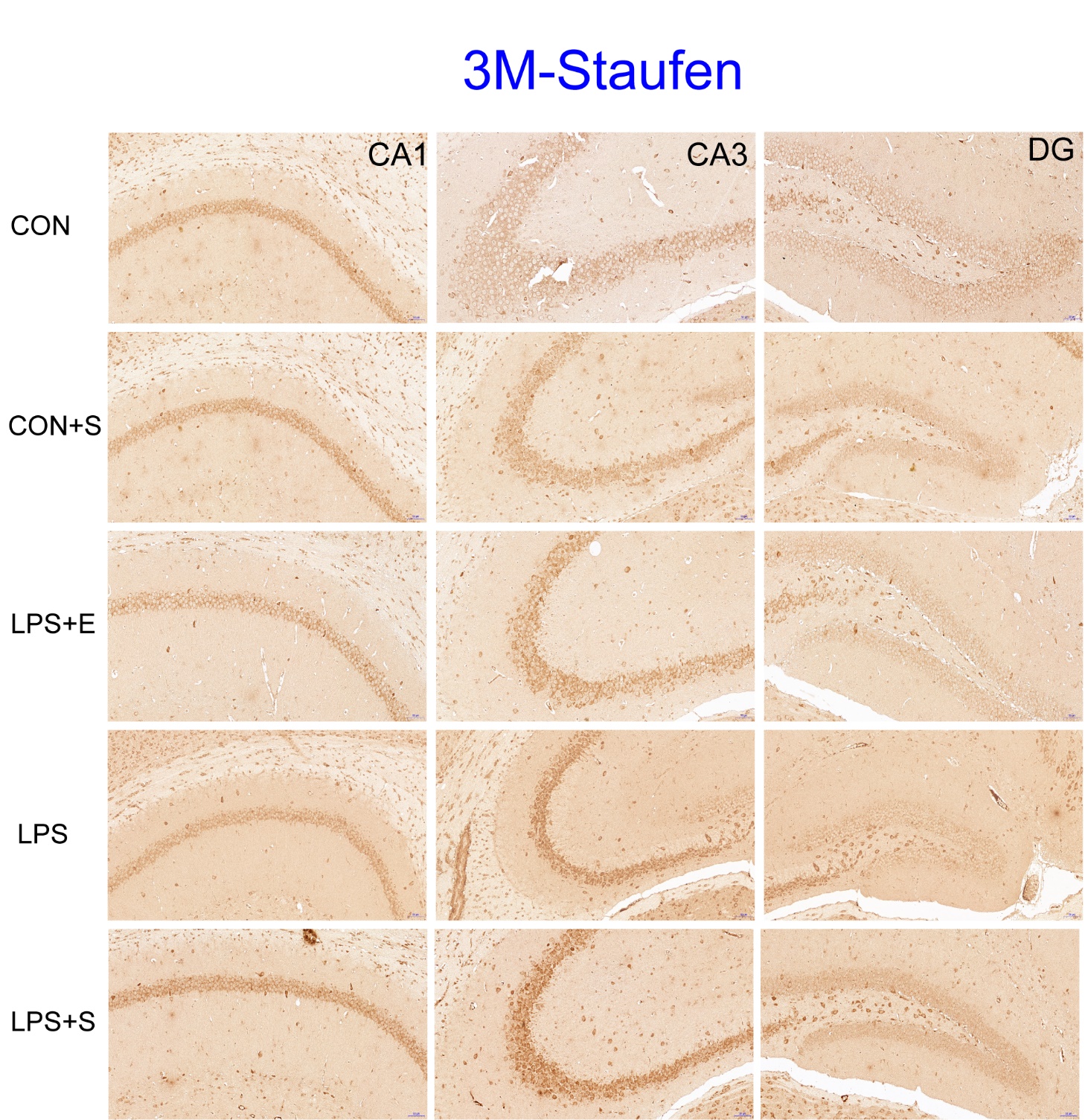


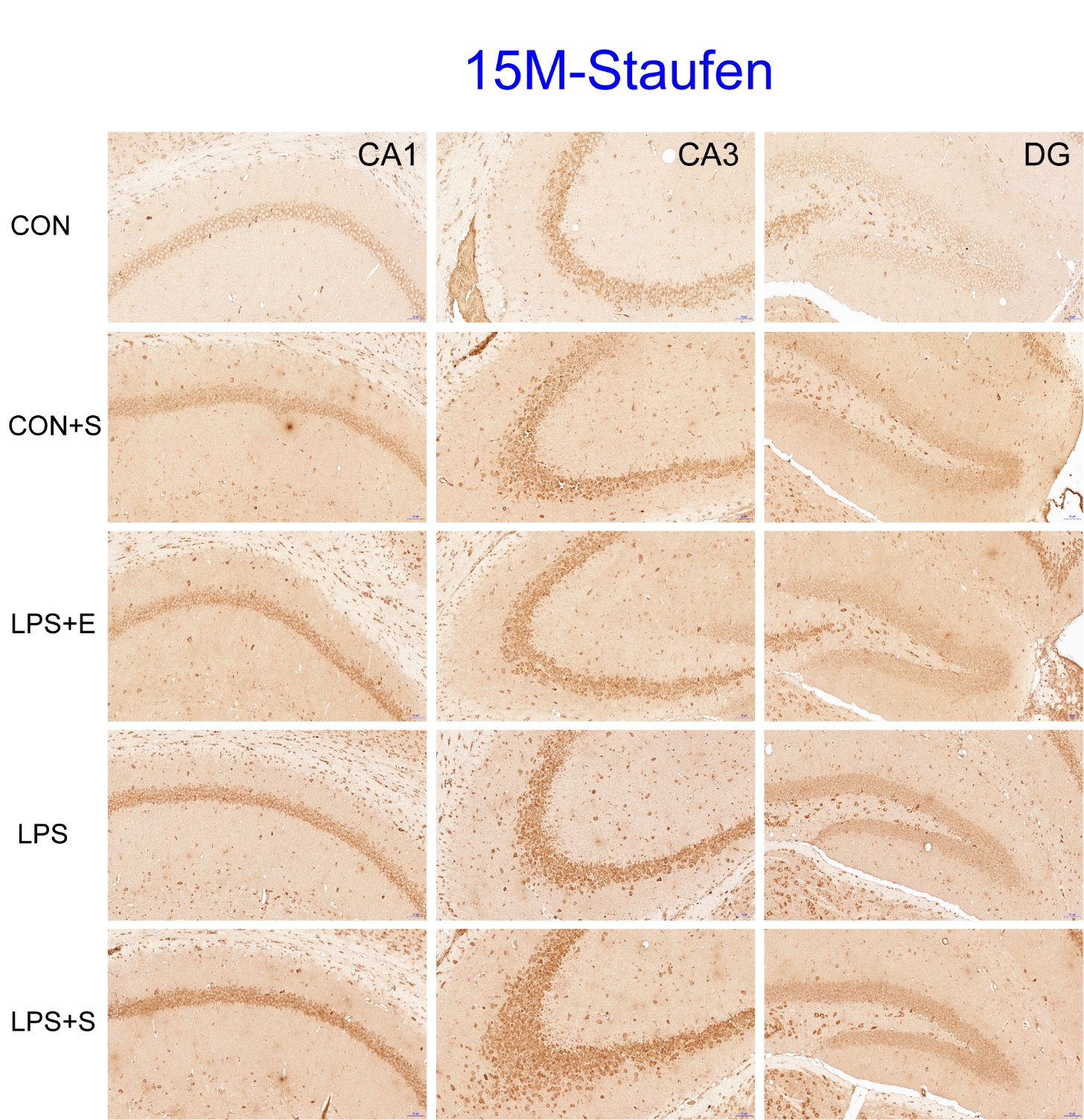


**Supplementary Figure 3.** Representative photomicrographs of Staufen immunolabeling in different hippocampal subregions in 3M (**A**) and 15M (**B**) mice. Scale bar = 50 µm. *N*=6 male mice/group. CA, cornu ammonis; DG, dentate gyrus; 3M, 3-month-old mice; 15M, 15-month-old mice; CON, untreated control group; LPS, lipopolysaccharide treatment group; S, group of mice exposed to stress; E, group of mice exposed to an enriched environment.


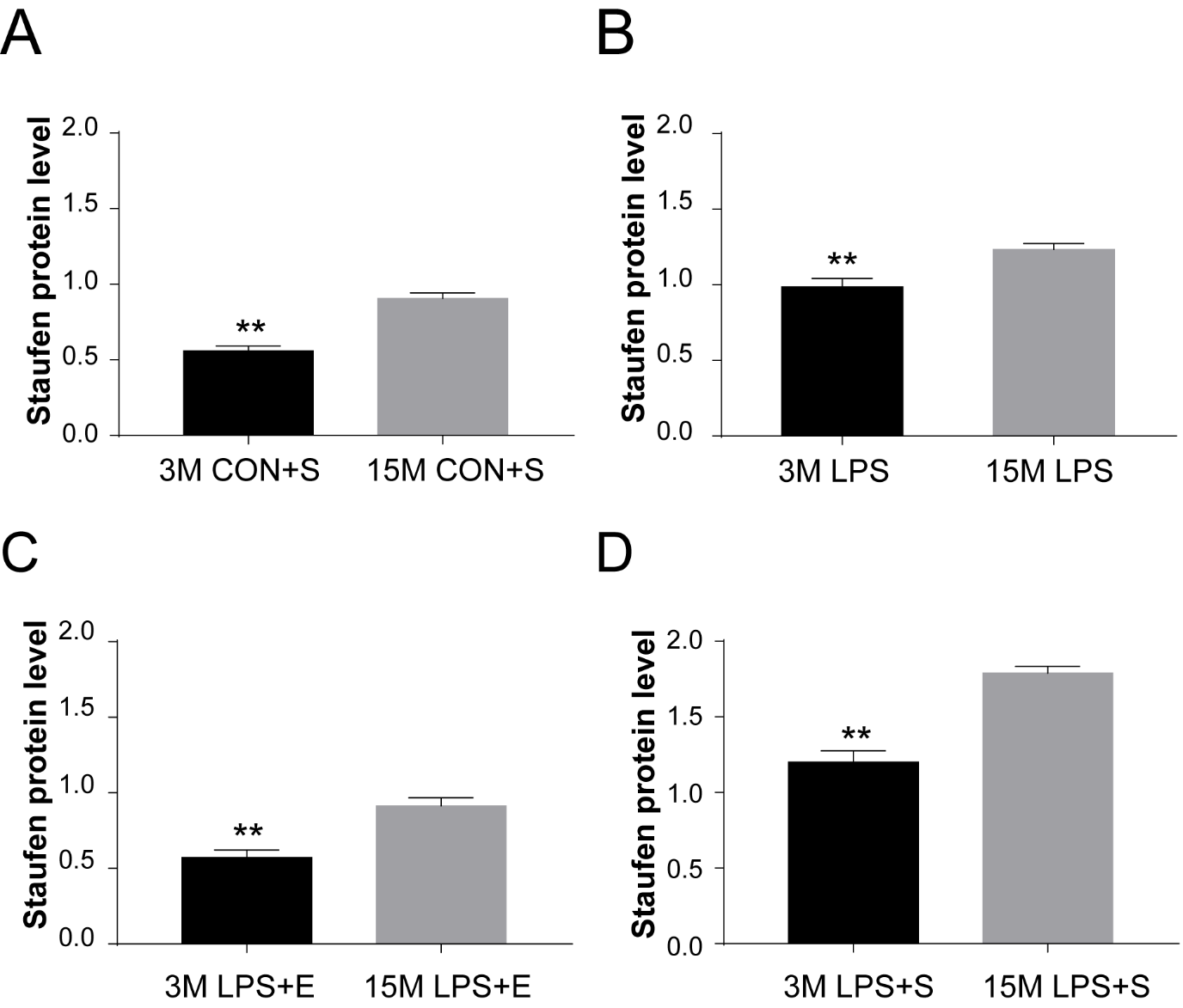


**Supplemental Figure 4.** The level of Staufen protein in mice undergoing different treatments (except for the CON group). The 15M mice exhibited higher levels of Staufen protein than 3M mice in the CON+S (**A**), LPS (**B**), LPS+E (**C**), and LPS+S (**D**) treatment groups. Error bars = SEM. *N*=6 male mice/group. **P* < 0.05, ***P* < 0.01 comparison between groups undergoing the same treatment. 3M, 3-month-old mice; 15M, 15-month-old mice; CON, untreated control group; LPS, lipopolysaccharide treatment group; S, group of mice exposed to stress; E, group of mice exposed to an enriched environment.


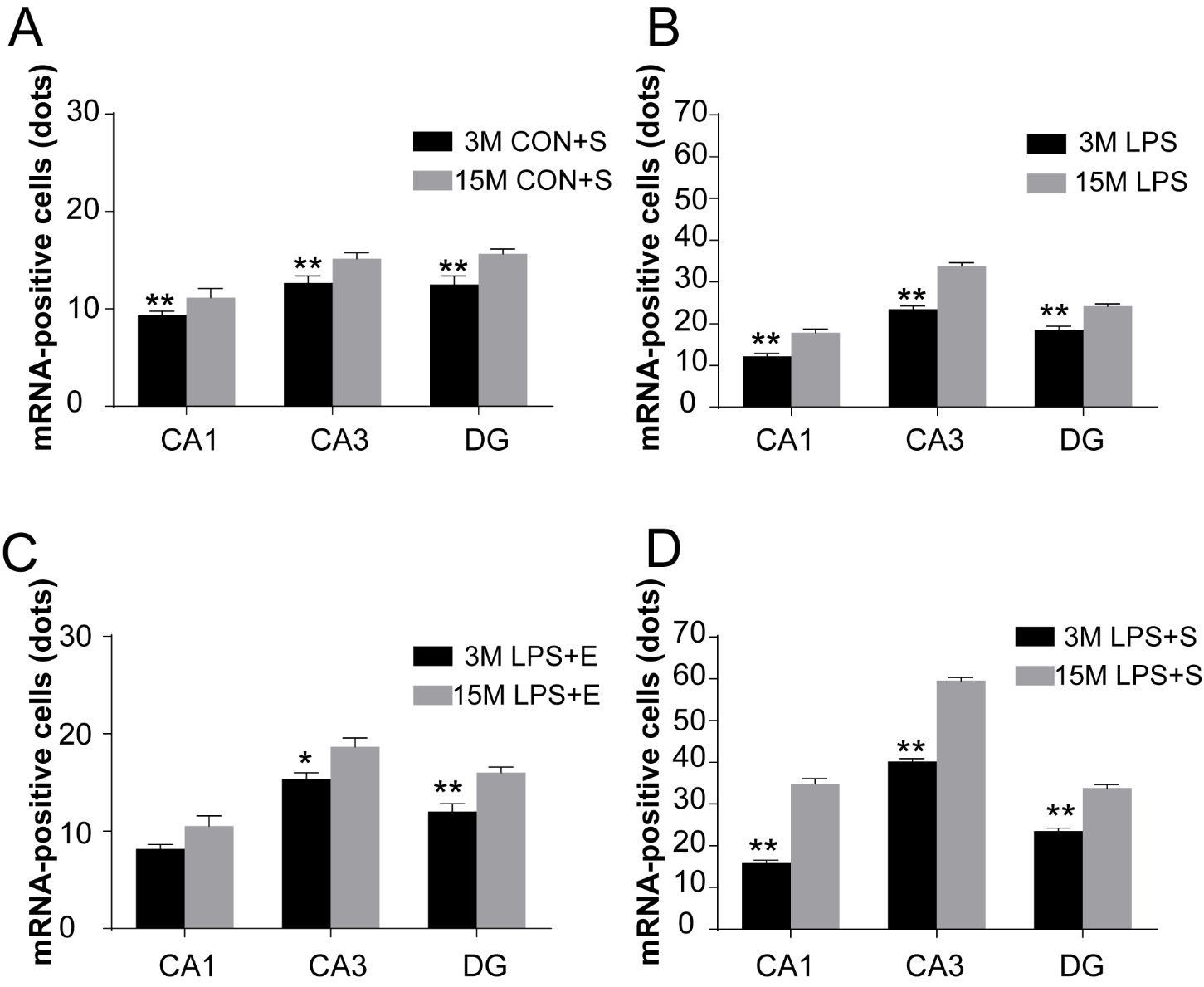


**Supplementary Figure 5.** The level of Staufen mRNA in different hippocampal subregions in CD-1 mice of both ages (except for the CON group). The 15M mice showed higher levels of Staufen mRNA in the CA1, CA3, and DG subregions than 3M mice in the CON+S (**A**), LPS (**B**), LPS+E (**C**), and LPS+S (**D**). Error bars = SEM. *N*=6 male mice/group. **P* < 0.05, ***P* < 0.01 comparison between groups undergoing the same treatment. CA, cornu ammonis; DG, dentate gyrus; 3M, 3-month-old mice; 15M, 15-month-old mice; CON, untreated control group; LPS, lipopolysaccharide treatment group; S, group of mice exposed to stress; E, group of mice exposed to an enriched environment.

**
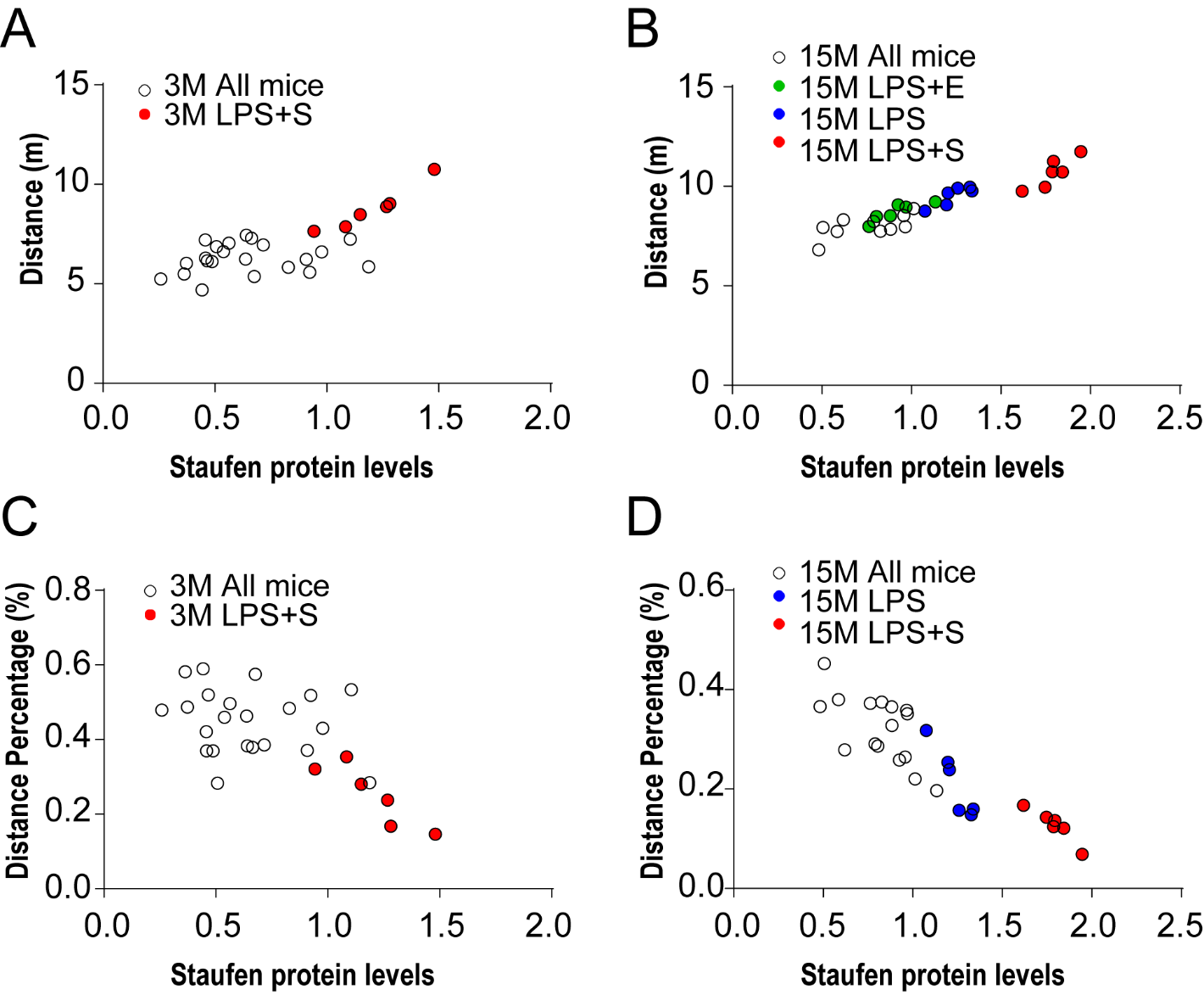
**

**Supplementary Figure 6A.** The correlations between hippocampal Staufen protein levels and learning and memory performance. **(A)** and (**B**) show the correlations between the learning swimming distance and hippocampal synaptic protein levels respectively in 3M and 15M mice. **(C)** and (**D**) show the correlations between the memory percentage of distance in the target quadrant of the Morris water maze (MWM) test and hippocampal synaptic protein levels respectively in 3M and 15M mice. (
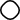
 all mice;
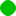
 LPS+E;
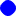
 LPS;
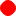
 LPS+S, the group which had the significantly correlation coefficients in the Table 1, *P* < 0.05). *N*=6 male mice/group. 3M, 3-month-old mice; 15M, 15-month-old mice; LPS, lipopolysaccharide treatment group; S, group of mice exposed to stress; E, group of mice exposed to an enriched environment.


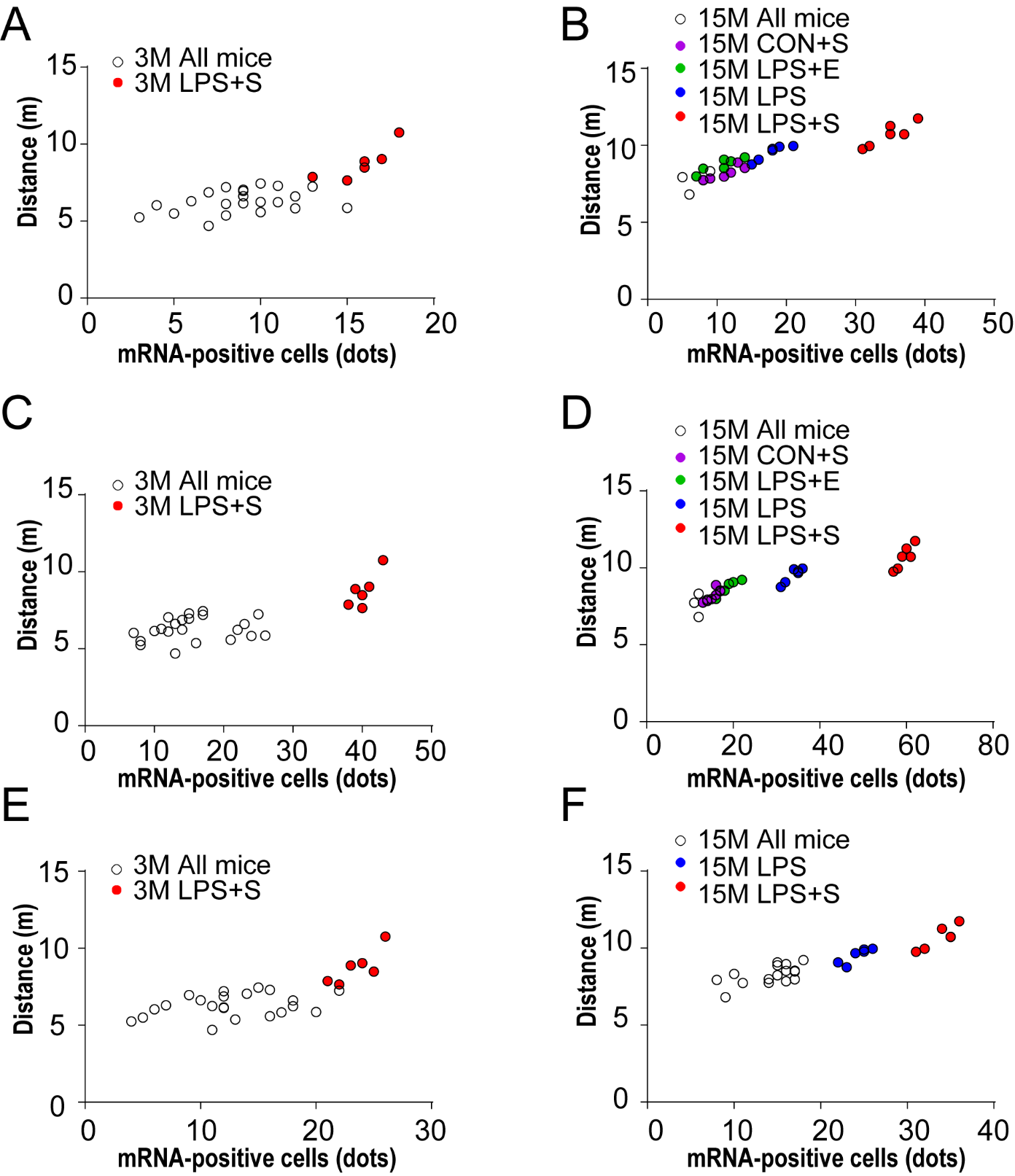


**Supplementary Figure 6B.** The correlations between the learning swimming distance of the Morris water maze (MWM) and hippocampal Staufen mRNA-positive cells in CA1 (**A** and **B**), CA3 (**C** and **D**), and DG (**E** and **F**) subregions in 3M and 15M mice. (
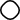
 all mice;
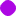
 CON+S;
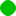
 LPS+E;
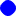
 LPS;
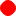
 LPS+S, the group which had the significantly correlation coefficients in the Table 2, *P* < 0.05). *N*=6 male mice/group. CA, cornu ammonis; DG, dentate gyrus; 3M, 3-month-old mice; 15M, 15-month-old mice; CON, untreated control group; LPS, lipopolysaccharide treatment group; S, group of mice exposed to stress; E, group of mice exposed to an enriched environment.

**
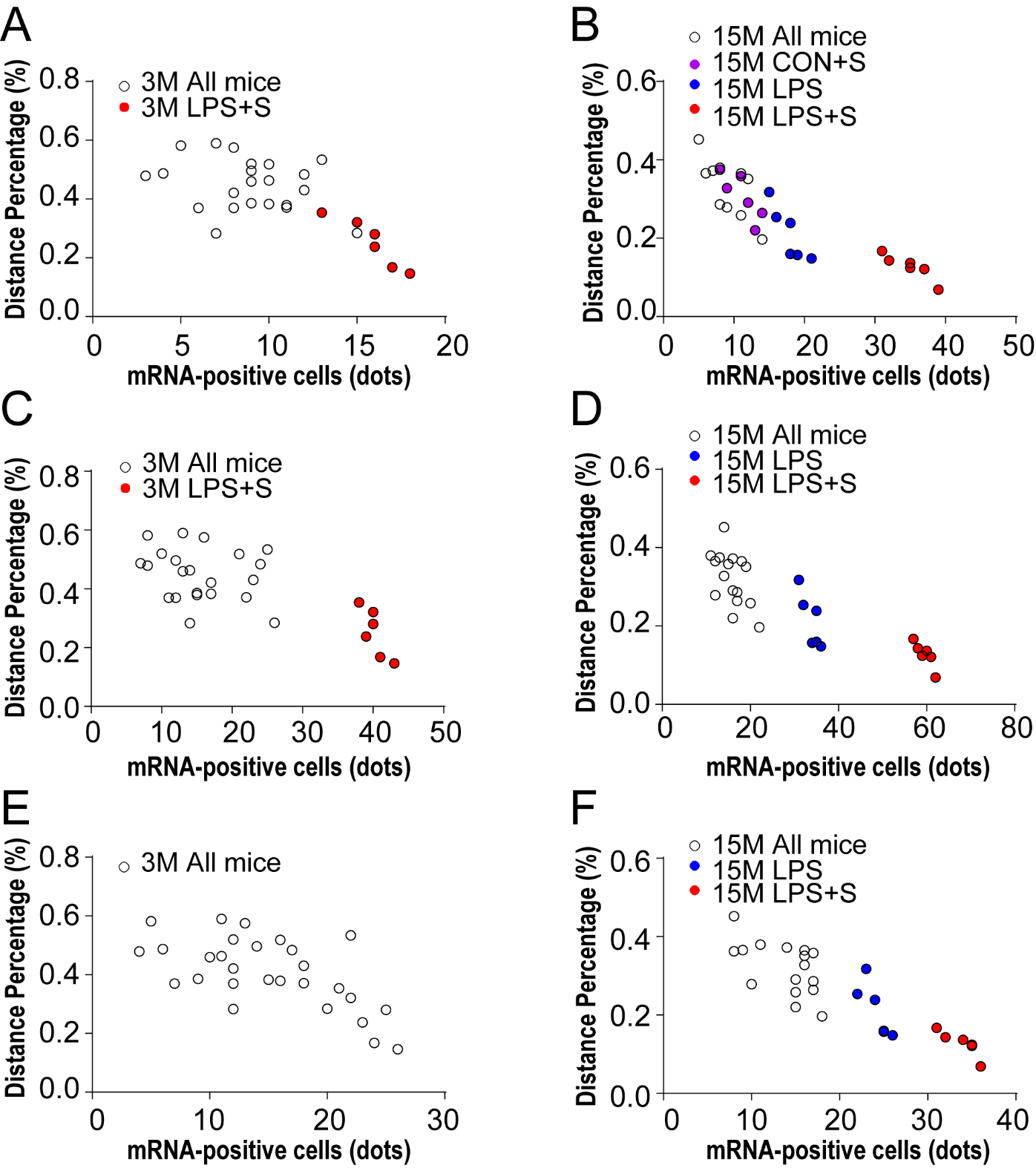
**

**Supplementary Figure 6C.** The correlations between the memory percentage of distance in the target quadrant of the Morris water maze (MWM) and hippocampal Staufen mRNA-positive cells in CA1 (**A** and **B**), CA3 (**C** and **D**), and DG (**E** and **F**) subregions in 3M and 15M mice. (
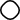
 all mice;
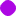
 CON+S;
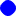
 LPS;
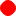
 LPS+S, the group which had the significantly correlation coefficients in the Table 2, *P* < 0.05). *N*=6 male mice/group. CA, cornu ammonis; DG, dentate gyrus; 3M, 3-month-old mice; 15M, 15-month-old mice; CON, untreated control group; LPS, lipopolysaccharide treatment group; S, group of mice exposed to stress.

**
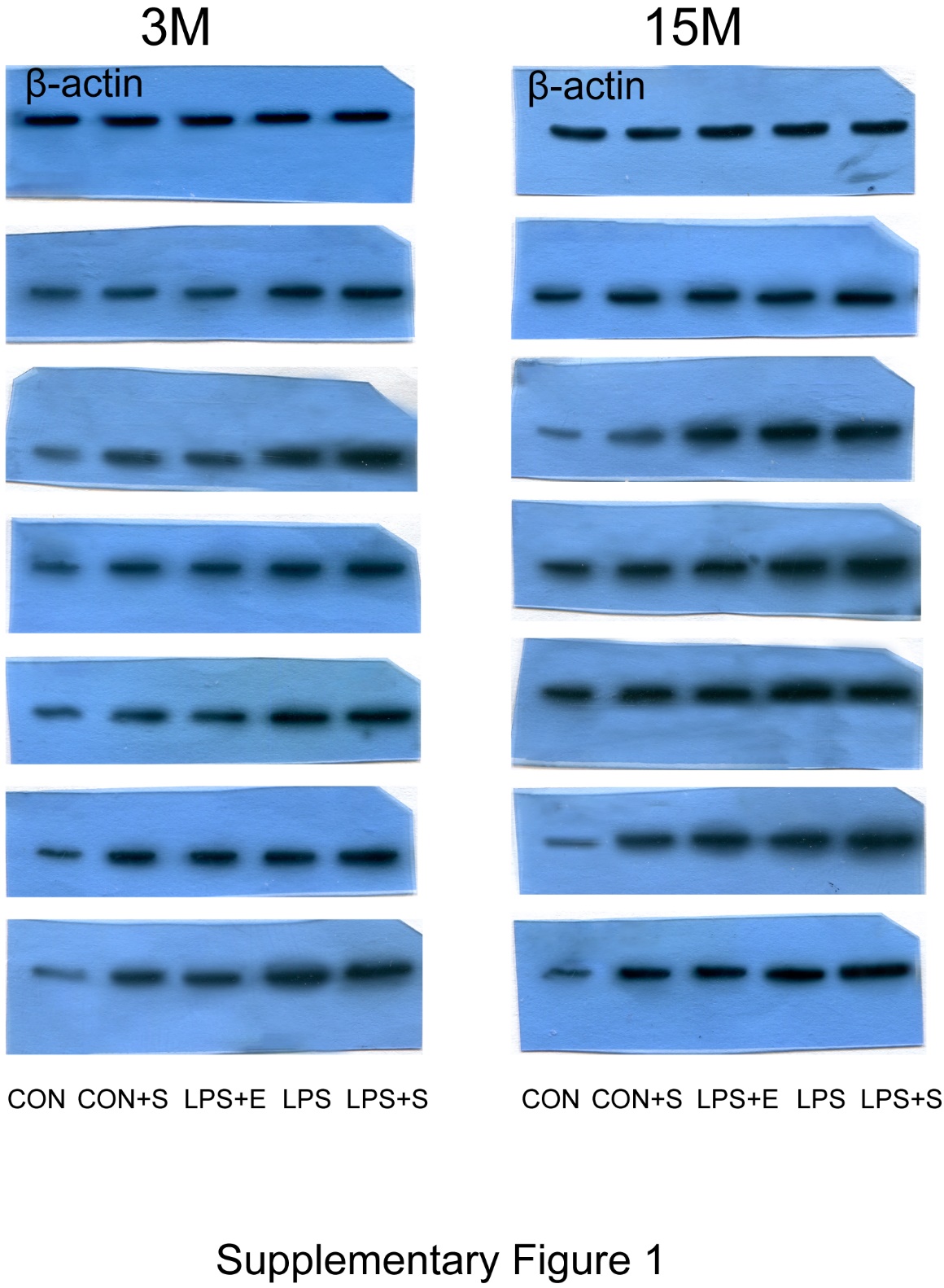
**

**Supplementary Figure 7.** Original western blotting images for the Staufen protein in CD-1 mice. Beta-actin was used as the control for each age. *N*=6 male mice/group. 3M: 3-month-old mice; 15M: 15-month-old mice; CON, untreated control group; LPS, lipopolysaccharide treatment group; S, group of mice exposed to stress; E, group of mice exposed to an enriched environment.
